# Optimizing Computer–Brain Interface Parameters for Non-invasive Brain-to-Brain Interface

**DOI:** 10.3389/fninf.2020.00001

**Published:** 2020-02-07

**Authors:** John LaRocco, Dong-Guk Paeng

**Affiliations:** Laboratory of Biomedical Ultrasound, Department of Ocean System Engineering, Jeju National University, Jeju City, South Korea

**Keywords:** interface, computer–brain, neuromodulation, non-invasive, temporal resolution, device portability

## Abstract

A non-invasive, brain-to-brain interface (BBI) requires precision neuromodulation and high temporal resolution as well as portability to increase accessibility. A BBI is a combination of the brain–computer interface (BCI) and the computer–brain interface (CBI). The optimization of BCI parameters has been extensively researched, but CBI has not. Parameters taken from the BCI and CBI literature were used to simulate a two-class medical monitoring BBI system under a wide range of conditions. BBI function was assessed using the information transfer rate (ITR), measured in bits per trial and bits per minute. The BBI ITR was a function of classifier accuracy, window update rate, system latency, stimulation failure rate (SFR), and timeout threshold. The BCI parameters, including window length, update rate, and classifier accuracy, were kept constant to investigate the effects of varying the CBI parameters, including system latency, SFR, and timeout threshold. Based on passively monitoring BCI parameters, a base ITR of 1 bit/trial was used. The optimal latency was found to be 100 ms or less, with a threshold no more than twice its value. With the optimal latency and timeout parameters, the system was able to maintain near-maximum efficiency, even with a 25% SFR. When the CBI and BCI parameters are compared, the CBI’s system latency and timeout threshold should be reflected in the BCI’s update rate. This would maximize the number of trials, even at a high SFR. These findings suggested that a higher number of trials per minute optimizes the ITR of a non-invasive BBI. The delays innate to each BCI protocol and CBI stimulation method must also be accounted for. The high latencies in each are the primary constraints of non-invasive BBI for the foreseeable future.

## Introduction

Non-invasive brain-to-brain interface (BBI) requires precision neuromodulation, device portability, and high temporal resolution to increase accessibility (Rao et al., 2014; Lee et al., 2017; Jiang et al., 2019). Two relevant non-invasive neuromodulation methods include transcranial focused ultrasound (TFUS) and transcranial magnetic stimulation (TMS) (Lee et al., 2018). Electroencephalography (EEG) offers high temporal resolution and sufficient spatial resolution for use in brain–computer interfaces (BCIs) with low-cost, consumer-grade headsets (Volosyak et al., 2010; Lebedev and Nicolelis, 2017). A non-invasive BBI can potentially operate with limited computational resources, but no work has yet investigated the potential limitations of a general purpose, non-invasive BBI (Lee et al., 2017; Rao, 2019, Toward neural co-processors for the brain: combining decoding and encoding in BCIs 2019).

Brain-to-brain interfaces have been demonstrated with both invasive and non-invasive methods in humans and animals. A BBI is a combination of a BCI and a computer–brain interface (CBI).

The reported benefits of a BBI as a “brainet” include enhancements in cognitive performance and learning rate (Pais-Vieira et al., 2015; Cinel et al., 2019). The accessibility of human BBIs has been previously constrained by CBIs, typically requiring highly invasive implanted devices, imprecise neurostimulation techniques, and immobile equipment (Ye et al., 2016; Martins et al., 2019). Developments in technology and software may offer alternatives to these existing barriers, but little analysis has been done on potential system data flow rates for non-invasive BBI. To contextualize brain-to-brain communication, therefore, the quantification of neural signals, a coordinate system, computer–brain information delivery parameters, and a communication format are required. Information delivery parameters will be addressed in depth.

Brain-to-brain interface systems are a combination of BCI systems and neurostimulation, and EEG-based BCI protocols are among the least invasive and lowest cost to implement (Volosyak et al., 2010; Mathe and Spyrou, 2016). EEG offers neurophysiological recording with high temporal resolution using a proven coordinate system. The International 10–20 system standardizes EEG electrode positions (Rao et al., 2014). The International 10–20 system calculates electrode positions relative to the distance between the nasion, the bridge of the nose, and the inion, the base of the skull. The center of this direct line is position CZ, from which all other electrode positions are calculated. The International 10–20 system is used in the current study to define EEG electrode and transducer placement (Yoo, 2018).

Electroencephalography has been extensively utilized in non-invasive BCIs, and four common EEG BCI protocols are motor imagery, covert speech, steady-state visually evoked potential (SSVEP), and P300 virtual keyboard protocols (Beverina et al., 2003; Schlögl et al., 2005; Sereshkeh et al., 2017). Common BCI protocols, such as SSVEP and P300 virtual keyboards, rely on visual and auditory feedback (Beverina et al., 2003). Such feedback may require a user to remove their attention from the task at hand, potentially lowering the efficiency of direct brain-to-brain communication. Prior non-invasive BBI systems did not require the patient to divert their eyes from the task at hand, as the feedback was integrated into the task itself (e.g., video game sprite movement) (Rao et al., 2014; Lee et al., 2017). Motor imagery and covert speech present examples of proven BCI protocols that do not require extraneous visual feedback, unlike the SSVEP and P300 speller (Schlögl et al., 2005; Sereshkeh et al., 2017). BCI systems using imagined movements and speech have been implemented with consumer-grade dry electrode EEG headsets, which have few electrodes and are susceptible to noise (Mathe and Spyrou, 2016).

A key metric for BCI performance is the information transfer rate (ITR) (McFarland et al., 2003; Schlogl et al., 2003). Measured in bits per trial or bits per minute (bpm), the ITR is a function of the speed with which a user can successfully send commands and receive responses from a properly calibrated system (Krausz et al., 2003; Blankertz et al., 2006; Thomas et al., 2013; Chen et al., 2014). ITR is a function of the number of classes and accuracy of a system, so a higher accuracy directly increases the ITR. For motor imagery-based EEG BCIs, an ITR value of 35 bpm was the highest reported value for years (Blankertz et al., 2006). However, that value was limited by the length of the trials, and ITRs of approximately three times that have been reported in recent years. Despite this, the trial length can still affect the ITR, so bits per trial is a more suitable measure in certain cases. A value of 1 bit/trial has been reported as a respectable value, regardless of trial length (Obermaier et al., 2001). A current standard for non-invasive BCI systems is approximately 100 bpm or less. Some groups have reported higher rates, which including one reporting a rate as high as 302 bpm (Lin et al., 2019).

Prior research has examined the viability of BCI systems partially, or automatically, adjusting the length of trials and epochs to improve ITR (Jin et al., 2019b). This work has been performed with common paradigms, such as motor imagery, the P300 speller, and SSVEP (Dimitriadis and Marimpis, 2018; Feng et al., 2018; Jin et al., 2019a). In addition, BCI feature selection can be similarly adjusted in response to each subject (Saha et al., 2019). A combination of a self-calibrating BCI, with automated feature optimization, could improve BCI performance, independent of subject (Saha et al., 2019). The primary advantage would be bypassing a time-intensive calibration session.

A poorly calibrated system requires more trials to achieve the correct classification and, thus, exhibits a lower ITR. Relying on EEG features that require a longer latency period, such as the P300, can lower the ITR. In the context of BBI and medical imaging, a longer stimulation period can similarly decrease the ITR (Lee et al., 2017). Higher ITRs have been reported for invasive BCI and BBI systems than for their non-invasive counterparts, but electrode coverage, protocol selection, algorithm design, and rapid neurostimulation may change this (Danilov and Kublanov, 2014). The neurostimulation process must also not compromise the successful operation of a BCI by introducing EEG artifacts.

Computer–brain interface is needed to close the loop for BBIs. Closed-loop neurostimulation software has existed for over a decade, especially for neural cell cultures and invasive systems. BCI systems close the loop through visual, tactile, or auditory feedback. However, these are insufficient for the purposes of non-invasive, high-precision neurostimulation (George and Aston-Jones, 2010; King et al., 2013). In two previous non-invasive BBI implementations, TMS and TFUS were used (Rao et al., 2014; Lee et al., 2017). However, TMS also directly interferes with EEG recordings, requiring extensive noise-filtering systems. TMS systems are currently large and bulky. As a mechanical system, TFUS can be utilized with lower requirements for power, space, and noise cancelation in real time (Sassaroli and Vykhodtseva, 2016). Therefore, TFUS and a non-visual protocol EEG BCI present a potential framework for a completely non-invasive, non-surgical, and portable closed-loop BBI headset.

Traditional ultrasound transducers utilize a piezoelectric crystal to generate an acoustic output. They require specific parameters for successful sonication, including input function, frequency, amplitude, power, and periodicity (King et al., 2013). In the case of TFUS, power and precision are closely managed to prevent intense pressure and cavitation from resulting in neural damage (King et al., 2013; Lee et al., 2018). The implementation of a TFUS transducer would closely follow the specifications of systems previously used for successful BBI (Lee et al., 2017).

One relative drawback with TFUS BBI systems has been the longer sonication time, such as the 0.5 s reported in previous examples. In contrast, TMS-based BBI has used a stimulation period of 1 ms when the entire stimulation sequence was two 1-ms pulses spaced 8 s apart for safety (Rao, 2019, Toward neural co-processors for the brain: combining decoding and encoding in BCIs 2019). In comparison, invasive neurostimulation systems are able to execute alternating periods of 50-ms recording and 50-ms stimulation for a total cycle time of 100 ms or less (Pais-Vieira et al., 2015; Rao, 2019, Toward neural co-processors for the brain: combining decoding and encoding in BCIs 2019). Previous invasive stimulation, TMS, and TFUS BBI systems have had low effective ITRs.

Computer–brain interface systems must account for both hardware delays and the latency of human awareness. Although hardware and software can be optimized, prior non-invasive BBI attempts have been constrained by existing technologies. TMS devices are too large and ungainly for mobile use. Piezoelectric transducers, used in TFUS, require water or hydrogel bonding. Power dissipation and controlling intracranial pressure are key safety concerns for neurostimulation. The 0.5-s sonication period used in previous TFUS BBIs was five times the minimum latency of human awareness, resulting in a lower ITR (Lee et al., 2017). Similarly, medical imaging requires specific target sequences constrained by transducer design. Whereas transducer technology may improve, other approaches may result in shorter, sharper sonication periods, such as using an array of smaller, lower-power transducers, or developments in ultrasound imaging (Jin et al., 2015). A rapid-firing, lower-energy transducer could ensure a higher ITR for TFUS in conjunction with optimized device communication. Regardless of the stimulation method, BBI systems rely on potentially different system architectures.

Not all BBI systems require identical architectures. Although BCI is a well-explored area in the literature, a few use-cases for CBI have been investigated. Four BCIs in well-documented areas have been considered (Beverina et al., 2003; Blankertz et al., 2006). One was a therapeutic neurostimulation or medical CBI, performed for patients that would otherwise rely on an invasive deep brain stimulation (DBS) device or other conditional neurostimulation stimulation for chronic conditions (Lee et al., 2018). Another was a consumer-grade system that was designed for use by gamers or hobbyists (Mathe and Spyrou, 2016). Another was a high-level research or clinical one, intended for use in hospitals, clinics, and devoted laboratories (Schlogl et al., 2003). Each had a different structure for processing incoming data, and each represented a closed-loop system architecture.

A therapeutic neurostimulation device could be a CBI device combined with EEG or another biosensor. The would be monitored by an onboard processor for signs of the condition or event (Peiris et al., 2011). For example, signs of a Parkinson’s tremor may initialize neurostimulation. Under these circumstances, there are only two states: the stimulation condition and the absence of the condition. Classifier accuracy, update rate, and stimulation accuracy are the most important parameters. The performance characteristics of such a system could also be relevant to drowsiness and fatigue detection systems proposed for use in certain industries, such as manufacturing and transportation (Peiris et al., 2011). Each window of incoming data would be monitored, so single-trial performance is key (Rao, 2019, Toward neural co-processors for the brain: combining decoding and encoding in BCIs 2019).

The second case is a consumer model that could be used by gamers, hobbyists, and even labs with specific needs (Pan et al., 2017; Pathirana et al., 2018; InteraXon, 2019). It could be realistically realized today by combining a consumer EEG headset with low-cost transducers. The gaming headset would have a higher number of states, corresponding to the game inputs. Just as BCI systems have used virtual keyboards, a gaming controller application may be likened to a virtual keypad. However, hobbyists and labs could adapt it as a full virtual keyboard. In both cases, the number of states is greater than two, and the system requires rapid responses. As data rapidly stream in, low system latency, a rapid update rate, and an optimized time cutoff are of great importance, so that even a single classification error can be rapidly compensated for Rao (2019), Toward neural co-processors for the brain: combining decoding and encoding in BCIs 2019).

The last case is a research system, comparable in performance to high-end medical EEGs rather than consumer headsets. The device would require an accurate classifier and reliable stimulation. Data, likely from many electrodes, would require rapid and accurate classification from the BCI. A research CBI requires reliable, low-latency stimulation. Such high-demand systems must be capable of integration with sensory inputs or diagnostic tools for experimental and medical work (Rao, 2019, Toward neural co-processors for the brain: combining decoding and encoding in BCIs 2019). A virtual keyboard is a common research BCI system, where a user selects from different characters or options on a screen (Beverina et al., 2003). In the context of BBI, it represents a system with a high number of classes. Protocols and findings from a research system may eventually be generalized for consumer or therapeutic CBI (Lee et al., 2017).

Non-invasive BBI systems have been successfully implemented and tested on humans with both TFUS and TMS (Rao et al., 2014; Lee et al., 2017). The architectures used were constrained by laboratory-based protocols and experimental implementation, which are impractical for other applications and difficult to replicate. To maintain an ITR comparable with that of existing BCI systems, a BBI requires a rapid stimulation method, low-latency communication, reliable stimulation, confirmation of stimulation, and the accurate classification of input signals. To date, no research has addressed optimizing these parameters in the context of non-invasive BBIs. The primary gap in the literature is the lack of CBI parameter consideration in the context of existing non-invasive BCI and EEG analysis. The purpose of this work is to simulate and optimize the necessary CBI parameters for a non-invasive EEG-based BBI system.

## Materials and Methods

### Overview

**FIGURE 1:**
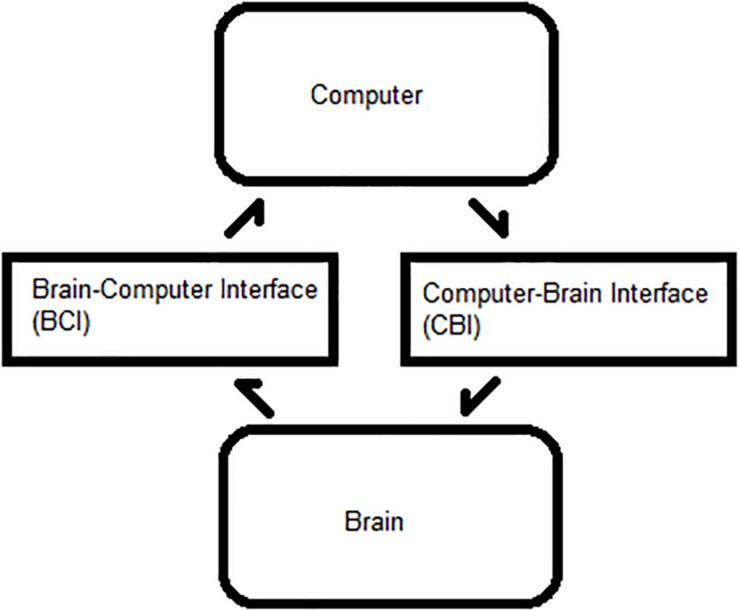
Process flow between a brain and computer. Bidirectional flow of processes between a user’s brain and an external computer system.

A BBI requires minimal delay between the BCI and CBI steps for each trial, as shown in Figure 1. The BCI reads and interprets commands, and the CBI translates them to a physiological output. This framework can be applied to BCI, BBI, therapeutic neuromodulation, and medical imaging. Optimizing the ITR facilitates system efficiency and is dependent on six primary BCI–CBI variables: classifier accuracy, number of states, update rate, system latency, stimulation reliability, and time cutoff (Chen et al., 2014; Rao et al., 2014; Krausz et al., 2003). Classifier accuracy, number of states, and update rate are BCI dependent, whereas the others are CBI implementation dependent. All six are simulated in MATLAB R2015a (Mathworks, 2015). During the BCI phase, the update rate is directly proportional to the ITR in bits per minute, but BCI optimization is a well-documented topic in the literature. CBI parameter optimization is less common.

In all three CBI system architecture cases, the time duration of a trial is a fundamental limitation on information transfer. BCI protocols, such as motor imagery, covert speech, SSVEP, and P300 virtual keyboards, typically require multiple seconds. Diagnostic protocols, such as those used for therapeutic cases, require the “trial” to be a window long enough to extract meaningful information. Thus, the condition-dependent diagnostic information determines window length (LaRocco et al., 2014). Similarly, the update rate must be sufficient for both real-time response and context-specific features.

Similarly, human awareness acts as another potential bottleneck. In order to impart a “real-time” experience, a BCI system must provide feedback within 0.1–0.2 s of a trial. For a BBI, the CBI process must also be concluded within this 0.1–0.2-s interval. TFUS and TMS, as previously employed, took 0.5 and 8.0 s, respectively. Although rapid neurostimulation is done to prevent brain damage, it is essential for a responsive system (Rao et al., 2014; Lee et al., 2017). Even with rapid stimulation, a poor connection or a failed trial can similarly increase system latency.

### Simulation Scope

As other researchers have thoroughly covered BCI parameters, such as classifier accuracy, update rate, and window length, the scope of the presented results was focused on CBI parameters (Krausz et al., 2003; Omary and Mtenzi, 2009; Jin et al., 2019b). The simulated BBI modeled the effective ITR, including window length, update rate, classifier accuracy, number of states, system latency, stimulation reliability, and time cutoff. Window length is the total time span covered by each trial. The update rate is how fast each window refreshes. The classifier accuracy is defined as the rate at which the system can assign the correct state to new trials. The number of states is defined as the discrete number of groups able to be selected through user action (or inaction). System latency is the total time required for the data to be processed and classified and for stimulation to be delivered. Stimulation reliability is the percentage chance that the initial stimulation will fail, requiring up to two more attempts. Time cutoff is a timeout threshold that precludes any delayed stimulus delivery if exceeded. A single simulated BBI, a binary-state medical monitoring system, was used owing to its relative simplicity.

### Parameter Computation

With the use of Eq. 1, the ITR was calculated in terms of bits per trial. It is a direct function of two variables: system accuracy (*P*) and the number of classes (*N*). For example, a two-class therapeutic CBI system with a nearly perfect (>99.99%) classifier would have an ITR of 1 bit/trial. In this case, the number of classes (*N*) was set to 2, and the accuracy (*P*) was set to 0.99999.

(1) $$I\,T\,R=l\,o\,g_{2}\,\left(N\right)+P\times l\,o\,g_{2}\,\left(P\right)+\left(1-P\right)\times l\,o\,g_{2}\,\left(\frac{1-P}{N-1}\right)$$

The trial length is a function of window length, update rate, system latency, stimulation failure rate (SFR), and time cutoff. The window length was the total duration, in seconds, of a trial. The update length was the refresh time, in seconds, of the system. The system latency was the total time, in seconds, required to complete a full “cycle” of the BCI–CBI “loop.” The SFR was the probability of neurostimulation not being provided or not having the intended effect, resulting in a break in the loop. The time cutoff was the time, in seconds, after which a failed stimulation would not be reported. To convert between bits per trial and bits per minute, window length *L* (in seconds) and window update rate *U* (in hertz) were required.

(2) $$I\,T\,R\,\left(\frac{b\,i\,t\,s}{m\,i\,n\,u\,t\,e}\right)=I\,T\,R\,\left(\frac{b\,i\,t\,s}{t\,r\,i\,a\,l}\right)\times L\times U\times 60\,\left(\frac{s\,e\,c\,o\,n\,d\,s}{m\,i\,n\,u\,t\,e}\right)$$

In the case of the example system, window length *L* was 1 s, and the update rate *U* was 100 ms. If the sampling window was 1 s long, with an update rate of 100 ms, it would have a total ITR of 600 bpm. This rate is far higher than conventional BCIs, but it is possible for a binary-state EEG-based therapeutic system. Based on documented BCI protocols like motor imagery, P300 spellers, covert speech, and SSVEPs, an ITR lower than 100 bpm is more likely (Beverina et al., 2003; Blankertz et al., 2006; Lin et al., 2019). The values for specific CBI parameters are detailed further in the literature (Krausz et al., 2003; Chen et al., 2014).

As discussed previously, the ITR is primarily a function of classifier accuracy, the number of classes or selectable outputs, total time per trial, and the number of trials per time period. Recorded and typical ITR values for different BCI systems were used as a baseline for the CBI estimates. Based on the operational requirements discussed previously, a medical monitoring CBI system is likely to have the highest ITR, owing to its short time window (1.0 s) and rapid update rate (0.1 s). The monitoring system represents a primarily passive high-accuracy system, such as an occupational drowsiness detector (LaRocco et al., 2014). The research CBI is likely to have a high ITR but may be constrained by longer window lengths owing to its large number of classes. The research system represents an experimental setup, such as an EEG, with over a hundred active channels and several options (Rao, 2019, Toward neural co-processors for the brain: combining decoding and encoding in BCIs 2019). A virtual keyboard CBI would likely have the highest ITR, as it is a research CBI with a large number of potential states. A virtual keyboard is often a P300 speller in past studies, although other paradigms have been reported (Beverina et al., 2003). The consumer CBI is likely to have low accuracy but a large number of classes and trials per minute. Consumer systems include hobbyist and gaming projects, although some have been used in an experimental context (Pan et al., 2017). Of the four typical BCI systems, the medical monitor has the highest ITR, and a higher number of trials than the other three, even at lower classifier accuracies.

Table 1 investigates high-accuracy (99%), medium-accuracy (75%), and low-accuracy (50%) cases for each BCI model. Given the current values in the literature, it is unlikely that the ITR for other protocols is as high as for medical monitoring. Although 600 bpm seems like a high rate, a skilled typist is capable of producing thousands of bits per minute of text (Beverina et al., 2003; Blankertz et al., 2006). Therefore, non-invasive BBI may be constrained by protocols and hardware, at least when compared with invasive options (Rao, 2019, Toward neural co-processors for the brain: combining decoding and encoding in BCIs 2019). For the remainder of the simulation, the medical monitoring/therapeutic case was used owing to its high ITR (Chen et al., 2014). However, the ITR estimates could be adjusted for other cases by retrieving the literature values for BCI parameters, such as accuracy, number of classes, and update rate (and thus, number of trials per minute).

**TABLE 1:** ITR for different EEG BCI applications.

	**(a) Medical monitor**			**(b) Consumer**		
Accuracy	0.5	0.75	0.99	0.5	0.75	0.99
No. of classes	2	2	2	5	5	5
Bits/trial	0	0.19	1	0.32	1.01	2.32
Trials/min	600	600	600	60	60	60
Bits/min	0	114	600	19.2	60.6	139.2

	**(c) Research**			**(d) Virtual keyboard**		

Accuracy	0.5	0.75	0.99	0.5	0.75	0.99
No. of classes	10	10	10	50	50	50
Bits/trial	0.74	1.72	3.32	1.84	3.43	5.64
Trials/min	30	30	30	30	30	30
Bits/min	22.2	51.6	99.6	55.2	102.9	169.2

*ITR, information transfer rate; EEG, electroencephalography; BCI, brain–computer interface.*

### Information Transfer Rate Optimization

The total classification time included a simulated delay based on window length, sampling size, and probability of stimulation failure. The delay accounted for the CBI aspect of the system and modeled the sending, delivery, and confirmation of a stimulation sequence on another individual. The delay, or latency, of the sequence depended on the connection speed, sonication or stimulation period, and confirmation of stimulus delivery. Three CBI variables were explored for their effects on ITR: latency, SFR, and timeout.

The latency represented the entire time required to send the stimulation command, initialize the system, stimulate, and confirm stimulation. In previous studies, it ranged from 100 ms to 1 s without connection delays (Rao et al., 2014; Lee et al., 2017). The SFR is the ratio in which stimulation delivery does not result in the desired electrophysiological change or otherwise fails to achieve the desired goal, breaking the BCI–CBI loop. An amplitude-based threshold detector on the person to be stimulated was used to confirm proper stimulation delivery. If that threshold was not met, then the stimulation was repeated. The SFR values investigated were 0, 5, 10, 25, 50, 75, and 100%. The SFR may increase the total latency if stimulation delivery is not confirmed. The last variable was the timeout threshold, or the total length of time after a failed stimulation until a command is given to repeat it. The timeout threshold must be at least as long as the latency period. It is possible for a timeout threshold to compensate for a higher SFR; thus, a range of values from 100 ms to 1.5 s was explored. The latency, SFR, and timeout depended on the CBI implementation, and they affected the ITR by changing the total time required per trial. Their total impact was more thoroughly examined by keeping constant the BCI-dependent ITR variables, such as accuracy and the total number of classes.

### Statistical Testing

The latency, SFR, and timeout were examined in the context of total ITR. However, the findings demonstrated the limitations of the system implementation on the effective ITR. As shown in Table 2, a mixed-model analysis of variance (ANOVA) with repeated measures was used to investigate the statistical significance of the different effects. Among the hypotheses tested were as follows:

**TABLE 2:** Repeated-measures ANOVA for primary CBI parameters.

	**SumSq**	**DF**	**MeanSq**	***F***	***p* Value**	***p* Val. GG**	***p* Val. HF**	***p* Val. LB**
(Intercept)	4,688,156.48	3.00	1,562,718.83	630.75	0.00	0.00	0.00	0.00
Latency	76,489.69	3.00	25,496.56	10.29	0.00	0.00	0.00	0.00
SFR	196,179.39	3.00	65,393.13	26.39	0.00	0.00	0.00	0.00
Timeout	22,668.90	3.00	7,556.30	3.05	0.03	0.08	0.08	0.08
Error	5,544,796.83	2,238.00	2,477.57					

*CBI, computer–brain interface.*

*Hypothesis 1*: A shorter latency improves ITR, even at high failure rates.

Rationale: Shorter latency periods mean more commands can be transmitted, enabling a faster “recovery” period after a failure. However, latencies that are too short may not provide additional benefit under highly unreliable stimulus delivery.

*Hypothesis 2*: A higher failure rate will lower ITR, but it may be stabilized by the timeout threshold selection.

Rationale: A higher failure rate means more time is needed for a stimulation cycle. Beyond a particular threshold, a properly selected timeout stabilizes the decline in ITR.

After ANOVA, a *post hoc* Tukey test was performed to compare specific effects.

## Results

### Overview

In practice, the maximum potential ITR was directly dependent on the BCI parameters: window length, classifier accuracy, and update rate. To better investigate the effect of the CBI parameters, a simple BBI was simulated. For the simulated therapeutic and medical monitoring BBI system, the configuration was updated at a rate of 0.1 s, so a 1-s window corresponded to a 10 trials per second. There were 600 trials per minute. The implemented system was a two-class problem. Assuming the number of classes *N* = 2 and Accuracy = 99.99%, inserting these values into Eq. 1 yields 1 bi/trial, which is the theoretical maximum under perfect operation. Obviously, the values would be lower in a realistic scenario. As shown in Table 1, multiplying this by 600 trials per minute, the theoretical maximum ITR becomes 600 bpm. Although this seems substantial, a trained user with a conventional keyboard and mouse can achieve several kilobytes per minute (Obermaier et al., 2001). As the medical monitoring system showed the highest ITR of the typical BCIs, its results were generalized to the other systems. Adjusting the ITR values for another system type required only scalar multiplication of the values from Table 1 and then inserting them into Eqs 1 and 2.

An ITR of 600 bpm is six times higher than the real-time BCI implementations of ∼100 bpm. Real-time BCIs required longer trial lengths and different update rates, so the value of 600 bpm can be seen as more reflective of a medical analysis or fatigue monitoring system than EEG-based BCI (Beverina et al., 2003; Chen et al., 2014). The performance value of 1 bit/trial, though, is well within the reported values in the literature for motor imagery BCI performance (Obermaier et al., 2001).

Across the entire dataset, the ANOVA yielded a *p* value < 0.001. However, the *post hoc* Tukey tests and the more in-depth analyses revealed a wider range of effect sizes for the experimental variables. The first hypothesis was that shorter latencies could improve ITR, even at high failure rates. The second was that optimal threshold selection could limit the decline in ITR at high failure rates.

### Hypothesis 1: Latency and Failure Rate

**FIGURE 2:**
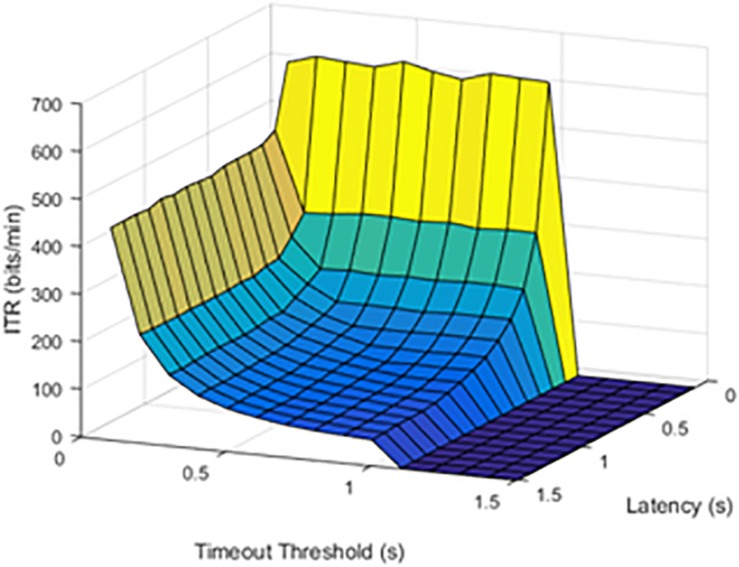
Information transfer rate (ITR) values at a 25% stimulation failure rate (SFR). The results show where stimulation command would fail 25% of the time.

**FIGURE 3:**
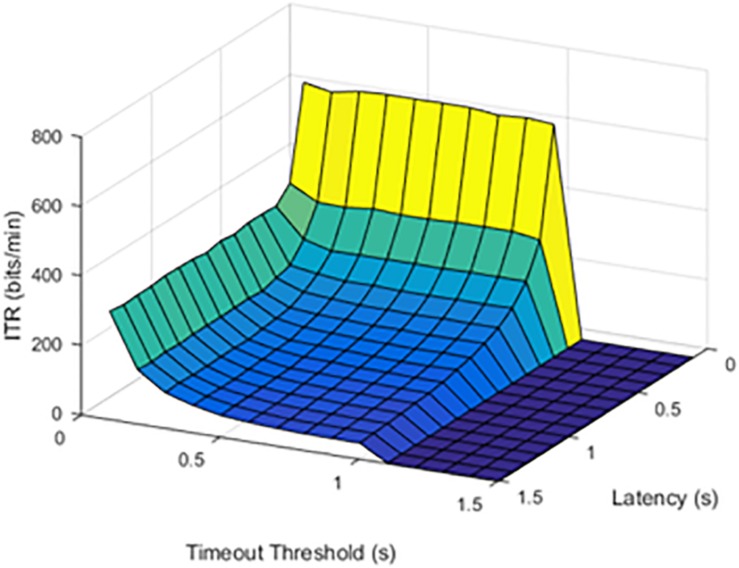
Information transfer rate (ITR) values at a 50% stimulation failure rate (SFR). The results show where stimulation would fail 50% of the time.

Although limited by the window size and refresh rate, latency was a limiting factor for the maximum possible ITR. Results were first visually inspected and compared in Figures 2, 3. An SFR of 25% affects the response pattern, as shown below.

When the failure rate was increased to 50%, the decrease in ITR continued.

The effect of increasing SFR was statistically significant, with a *p* value of 0.0092. Although a failure rate of 25% decreased the ITR, higher failure rates caused a significant drop in ITR.

### Hypothesis 2: Timeout Threshold Optimization

**FIGURE 4:**
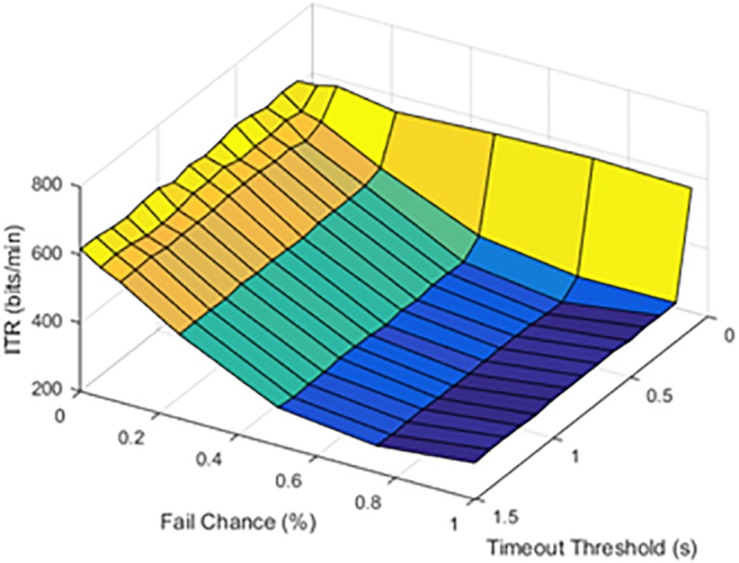
Information transfer rate (ITR), chance of failure, and cutoff threshold at a latency of 0.1 s.

The timeout thresholds and latency values were contrasted with each other. The results for a latency of 0.1 s were plotted in Figure 4.

**FIGURE 5:**
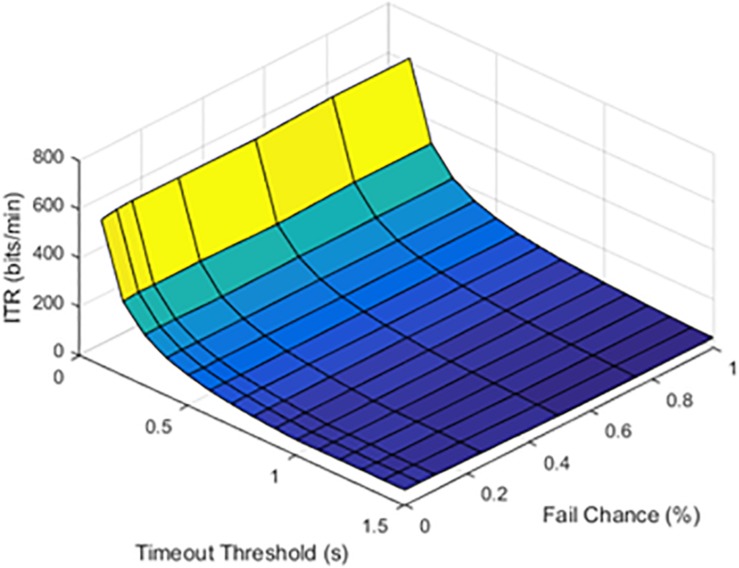
Information transfer rate (ITR), chance of failure, and cutoff threshold at a latency of 1.0 s.

At lower latencies, the temporal threshold of 0.1 s ensured an increase in ITR, even at high SFRs. As shown in Figure 5, the latency value of 1.0 s was shown as an example of higher latency values.

The results from timeout thresholds and failure rates were compared, resulting in a *p* value of <0.001. With longer latency periods, the system was unable to recover from a failed stimulation delivery. The optimal parameter selection, as shown in prior figures, could prevent dramatic declines in the ITR.

### Findings

Both hypotheses were confirmed. With parameter optimization, the system was capable of reaching ITR values close to the theoretical maximum. The selection of an optimized cutoff threshold can preserve ITR, even with an unreliable stimulation system. However, these results are highly dependent on the evaluated system. A realistic BBI would be more complex than a binary medical monitoring system, and the ITR would primarily be constrained by the number of trials per minute.

## Discussion

### Overview

The simulated results revealed the optimized parameter ranges. Shorter latency periods resulted in a higher ITR, limited by a sliding window update rate of 100 ms. The system was able to function, even with an SFR as high as 25%. For a temporal cutoff, a threshold of 100–200 ms was the most efficient for slowing the drop in ITR. Although an ITR of 600 bpm and a window update of 100 ms are far lower than those of a user with a conventional keyboard and mouse and other EEG BCIs, they are achievable with a low-cost, hobbyist EEG headset in the context of a two-class monitoring system (Obermaier et al., 2001; Pan et al., 2017). When CBI and BCI parameters are compared, the CBI’s system latency and timeout threshold should be reflected in the BCI’s update rate. In the simulated system, the highest ITR corresponded to the lowest latencies and timeout thresholds, even at high SFRs.

### Significance

The simulated BBI indicated that optimized parameters on both the CBI and BCI sides were required for proper function. Although a non-invasive BBI is an appealing concept, it is limited both by the length of the EEG necessary for proper BCI function and by the current limitations of non-invasive neurostimulation technology. Although low-bandwidth EEG headsets are limited compared with clinical or research systems, 100 ms was sufficient for “real-time” performance, at least in the context of certain monitoring tasks. When rapid stimulation is required, TMS may be required, whereas TFUS may be used when stimulation can take place over a period 0.5 s or more (Rao et al., 2014; Lee et al., 2017). Rapid and reliable stimulation is required for optimized BBI design, and a confirmation system may ensure that reliability. Similarly, a timeout value ensures the system is not “bogged down” by executing outdated commands. This suggests BBI systems may benefit from faster sampling windows, so as to maximize the number of trials in a given period. A higher ITR translates into a faster response time for a commercial BBI, faster character selection on a virtual keyboard, and so on. However, the longer response times inherent in existing BCI protocols also constrain the ITR (Beverina et al., 2003; Krausz et al., 2003; Chen et al., 2014).

Recombination of existing BCI protocols and CBI methods could potentially mitigate a lower effective ITR. For example, the BCI protocol could work directly with the CBI-stimulated brain region. A self-calibration or self-optimizing BCI paradigm could also be implemented (Dimitriadis and Marimpis, 2018). Such arrangements could also potentially compensate for the interference between the CBI and BCI, such as EEG electrodes and TMS loops. Although TFUS may lack the EEG interference of TMS, the transducer characteristics determine the effective stimulation parameters. In a previous study, a motor-imagery BCI was combined with TFUS (Lee et al., 2018). Covert imagery could potentially work in a similar arrangement for a BBI independent of visual stimuli. Direct integration of CBI into visual stimuli could greatly alter the dynamics of a BBI, such as in the case of a P300 or SSVEP paradigm (Chen et al., 2014). Such investigation would require extensive experimental results.

### Future Work

The primary limitation in the current study was the lack of experimental data. The physical implementation of a BBI is required to confirm simulation findings. Simulation values were based on the existing literature. The optimization of specific TFUS or TMS stimulation parameters would be required for future implementation of a real-time BBI. Similarly, existing TFUS and TMS both rely on high-resolution medical images to determine stimulation targets, so the automation of target identification and selection is required to bring non-invasive, self-optimizing BBI systems out of the lab. The inclusion of self-optimizing BCI systems could also be evaluated, both *in silico* and experimentally (Feng et al., 2018; Jin et al., 2019b). Furthermore, the development of a safe, rapid (<0.1 s) neurostimulation system would enable the performance of a non-invasive system to match an invasive one. Beyond the CBI parameters investigated, the size, precision, and power requirements of neurostimulation technology currently constrain non-invasive BBI.

## Conclusion

Brain-to-brain interface systems combine BCI and CBI systems in a closed loop for each user. Values from the existing literature were used to simulate parameters from BCI classifiers and CBI systems. The BBI systems required a fast ITR to compensate for low bandwidths, which was a direct function of the accuracy and the number of classes. In particular, a binary-state therapeutic monitoring system with a 1-s sampling window updated at a rate of 10 Hz was modeled. A rate of 1 bit/trial was used as the standard ITR value, which reached a value of 600 bpm in a two-class medical monitoring system. With the CBI latency kept constant at 100 ms, and a cutoff time of no more than 200 ms, a high ITR was retained, even with a SFR up to 25%. These findings suggest that CBI latency and timeout should be reflected in the update rate of a BCI to maximize the number of trials. The number of trials is directly proportional to the ITR. The delays innate to each BCI protocol and CBI stimulation method must also be accounted for. The high latencies of each are the primary constraints of non-invasive BBI for the foreseeable future.

## Data Availability Statement

The datasets can be found at: https://github.com/javeharron/bcidemo.

## Author Contributions

JL performed the simulations. D-GP provided facilities and support.

## Conflict of Interest

The authors declare that the research was conducted in the absence of any commercial or financial relationships that could be construed as a potential conflict of interest.
